# Evaluation of a care pathway for older adults presenting with nonspecific complaints at the emergency department: a before-and-after study

**DOI:** 10.1007/s41999-025-01226-8

**Published:** 2025-05-07

**Authors:** M. G. A. M. van der Velde, M. A. C. Jansen, F. Derkx-Verhagen, I. P. B. Tournoij, F. S. Jonkers, H. R. Haak, M. N. T. Kremers

**Affiliations:** 1 Department of Internal Medicine, Máxima MC, De Run 4600, 5504 DB Veldhoven, The Netherlands; 2 Department of Health Services Research, and CAPHRI School for Public Health and Primary Care, Aging and Long Term Care Maastricht, Maastricht, the Netherlands; 3 Netwerk Acute Zorg Brabant, Tilburg, the Netherlands; 4 Emergency Department, Máxima MC, Veldhoven, the Netherlands; 5 Department of Geriatrics, Anna Hospital, Geldrop, the Netherlands; 6 Emergency Department, Erasmus University Medical Center, Rotterdam, the Netherlands

**Keywords:** Emergency medicine, Nonspecific complaints, Older adults, Care pathway

## Abstract

**Purpose:**

Managing older patients presenting at the Emergency Department (ED) poses challenges due to their predisposition to present with nonspecific complaints (NSC). With an ageing population and ED overcrowding, significance of NSC is expected to grow. Therefore, we implemented a structured care pathway to address the complexity of NSC presentations. The aim of this study is to evaluate the effectivity of this care pathway.

**Methods:**

We conducted a before-and-after study to evaluate the NSC care pathway, which included risk stratification, standardized assessment and diagnostic measurements. A control group receiving standard care was established in two hospitals (hospital 1 and 2) before implementation. Patient enrollment occurred from April 2021 to November 2024. Outcomes included length of stay in the ED (LOS-ED) and in hospital (LOS-H), revisits, diagnostic completeness (i.e. 100% agreement between all ED and hospital discharge diagnoses) and perceived quality of care.

**Results:**

In total, 164 control and 235 intervention patients were included in this study. Median LOS-ED and LOS-H did not show significant differences between the control and intervention patients. Implementation of the care pathway showed non-significant trends towards improved diagnostic completeness (47.1% vs 37.2%, p = 0.096). This might be associated with the non-significant observed decrease in 30-day readmissions (7.5% vs. 12.7%, p = 0.256). Patient-reported outcomes indicated a positive experience with the quality of care.

**Conclusion:**

The care pathway did not improve LOS-ED and LOS-H as hypothesized, possibly due to logistical barriers and patient variability. However, trends suggested improved diagnostic completeness and fewer 30-day readmission rates. Further research initiatives are needed to enhance patient outcomes and care for NSC patients.

**Trial registry number:**

NL8960, date 9–10-2020.

**Supplementary Information:**

The online version contains supplementary material available at 10.1007/s41999-025-01226-8.

## Key summary points

**Aim:**

Management of older patients presenting to the Emergency Department (ED) with nonspecific complaints (NSC) is challenging. Therefore we implemented a structured care-pathway aiming to optimise diagnostics and treatment of this vulnerable population.

**Findings:**

NSC-patients showed increased comorbidities, polypharmacy and frailty. The implementation of the care-pathway showed non-significant trends towards improved diagnostic completeness, which might be associated with the reduced rate of 30-day readmissions. Patient reported outcomes measured in the intervention group, indicated a positive experience with the quality of care.

**Message:**

NSC-patients are a complex ED-population. The NSC-pathway showed trends towards improved diagnostic completeness and reduced 30-day readmission rates, but did not improve LOS-ED and LOS-H. Adaptation of the NSC-pathway could enhance effective and efficient care for this population.

**Supplementary Information:**

The online version contains supplementary material available at 10.1007/s41999-025-01226-8.

## Introduction

Management of older patients presenting at the emergency department (ED) poses a challenge due to their predisposition to present with poorly defined symptoms. These symptoms, such as ‘not feeling well’, unexplained falling, or being unable to cope with usual daily activities, are often referred to as nonspecific complaints (NSC) [1, 2]. The definition of NSC has not yet been formally established. However, most commonly, the description of Nemec et al. is used, which describes NSC as ‘complaints that are not part of a set of specific complaints or signs, or where an initial working diagnosis cannot be definitely established’ [3]. Given the continuous growth of the population of older patients seeking acute care, the significance of NSC gains importance [1].

Research has shown that patients with NSC are a vulnerable population with distinct characteristics in comparison to patients presenting with specific complaints (SC). Patients with NSC show a higher prevalence of comorbidities, polypharmacy and frailty [4, 5]. Combined with problems in functional status or communication, these factors lead to under-triage, increased complexity in the diagnostic process and a broad differential diagnosis, ranging from social issues to life-threatening conditions [4, 6, 7]. Consequently, this results in a high proportion of incorrect diagnoses and a great variety of discharge diagnoses in this population [1, 3, 8, 9]. Furthermore, patients with NSC often leave the ED with health issues that go unrecognised and untreated. This not only results in longer ED stays, but also leads to higher admission rates and a greater risk of adverse outcomes, including mortality [7, 10–12].

Addressing the needs of older patients with NSC is notably challenging due to a lack of standardised assessment protocols for these complex complaints. Various initiatives, for example geriatric screening at the ED, geriatric EDs or direct transfers to the geriatric ward, have been developed to enhance organisational and process structures within the ED [13–15]. However, accurately identifying patients at the highest risk for adverse outcomes remains difficult, leading to infrequent use of these interventions in practice. A comprehensive and structured approach is essential to effectively address the multifaceted nature of NSC presentations.

We proposed an integrated care pathway aimed at structuring and streamlining acute care for patients with NSC from arrival at the ED until discharge [16]. This structured approach involved risk stratification, systematic assessment of the patient and standard diagnostic measurements to ensure appropriate and timely interventions. We hypothesised that the NSC care pathway will lead to good perceived quality of care and reduce LOS-ED. Additionally, we expected that implementing this pathway might lead to a reduction in hospital length of stay (LOS-H), lower 30 day mortality and decrease (re-)admission rates. The aim of this study is to evaluate the effectivity of this care pathway after implementation, measured by the length of stay at the ED (LOS-ED), length of hospital admission (LOS-H), accuracy and completeness of (working) diagnoses and perceived quality of care.

## Methods

### Study design and setting

A detailed overview of the trial protocol, including the specifics of the care pathway, has been published previously [16]. The study initially aimed to include six hospitals in the province of Noord-Brabant, the Netherlands, all of which had initially agreed to participate. However, following the COVID-19 pandemic and the resulting delay of the study, only three ultimately confirmed their participation due to logistical challenges or concurrent research being conducted in the ED. As a result, a longitudinal parallel cohort trial (i.e. before-and-after study) was conducted in these three hospitals. However, one hospital withdrew mid-study due to logistical challenges, including understaffing and high workload from ongoing personnel issues, resulting in two participating hospitals. Of these, one is a general hospital, and the other is a teaching hospital.

### Study population

Older patients (≥ 70 years) with NSC referred to the ED by a general practitioner (GP) or elderly care physician (ECP) were screened for inclusion. Self-referred patients were eligible if triage at the ED confirmed NSC, provided they arrived at the ED between 8 AM and 8 PM. Patients referred by their known GP or ECP during working hours (8 AM–5 PM on weekdays) were eligible if they also arrived at the ED between 8 AM and 8 PM. All patients, whether self-referred or GP/ECP referred, were eligible for inclusion if they met the following criteria: (1) required ED admission, (2) were aged ≥ 70 years and (3) presented with an NSC.

NSC were divided into five referral categories: 1) somatic problems, such as weakness, not feeling well, change in nutritional status or unexplained weight loss; 2) an increased demand of care, such as loss of independency, a necessity for starting home care or for change in the living situation (such as admission to a care home), not indicated previously; 3) cognitive problems, such as disorientation, changes in behaviour or abrupt cognitive decline; 4) a decline in functional status, such as loss of mobility; and 5) unexplained falls, not related to extrinsic factors.

### Data collection

We conducted a before-and-after study to evaluate the NSC care pathway. Before implementation, each hospital included its own control group. The method of control group selection differed between hospitals due to variations in data access and infrastructure.

In hospital 1, control patients were included retrospectively by using a graphic user interface data mining tool with text-mining features (CTcue, version 3.0)[17]. CTcue allows for structured searches within unstructured electronic health record (EHR) data, enabling efficient identification of eligible patients based on predefined inclusion and exclusion criteria, study parameters and end points. This process ensured anonymous data retrieval, eliminating the need for informed consent.

In hospital 2, retrospective data retrieval via CTcue was not feasible. Instead, control patients were selected prospectively by the emergency physician (EP) before the NSC care pathway was implemented. The EP actively identified eligible patients in real time, registering their pseudonymised data for later analysis. Similar to hospital 1, informed consent was waived due to the anonymous nature of data collection.

Following implementation of the care pathway, eligible patients were provided with written patient information upon presentation at the ED and subsequently asked for informed consent. Moreover, they were asked to complete a questionnaire regarding patient satisfaction, known as the Patient-Reported Outcome Measurement-Acute Care (PRM-AC) [18].

### NSC care pathway

Before the implementation of the care pathway, patients received standard care. Upon arrival, they underwent triage, followed by history taking and physical examination. Frailty screening was not routinely performed, and there was no standardised approach for screening across the multiple domains of the comprehensive geriatric assessment (CGA); both were left to the discretion of the attending physician. Diagnostic tests were ordered according to the physician’s and nursing staff's judgement.

In contrast, the care pathway offered standardised care for patients with NSC [16]. Upon their arrival at the ED, patients were triaged and assessed for frailty in both cognitive and functional domains using the APOP screener [19]. In addition to solely somatic history taking, physicians conducted screening across various domains in line with the CGA [20]. NSC patients received standard diagnostic measurements, including an electrocardiogram, urinalysis, chest radiography, bladder ultrasound and comprehensive blood tests. Further diagnostic tests could be performed according to the physician’s choice.

The care pathway aimed to provide more systematic, standardised and comprehensive care, ensuring that frailty and multiple health domains were consistently assessed and addressed.

Assignment of the proposed dedicated ED nurse to oversee the care process for each patient in the care pathway was deemed unfeasible in participating hospitals due to personnel issues.

### Outcome measures

The primary outcomes were LOS-ED, as it reflects efficient care delivery, which is increasingly important given the challenges of overcrowding, and patient-perceived quality of care in five domains to assess patient centredness. Secondary outcomes included LOS-H, discharge destination, frequency of revisits/readmissions, as well as 30-day and 90-day mortality. Additionally, we assessed the effectivity of the pathway by evaluating the accuracy and completeness of the initial patient evaluation. Accuracy was assessed by comparing the working diagnosis at ED discharge with that in the hospital discharge letter after admission. Diagnosis was accurate when there was a 100% match between the working diagnosis at ED discharge and hospital discharge. Completeness of diagnoses was evaluated by comparing all diagnoses recorded by the attending physician in the ED with those documented by the discharging physician in the discharge letter. The level of agreement between these diagnoses was used to assess improvements in the diagnostic process. Completeness was defined as a 100% match between all diagnoses at ED discharge and hospital discharge. This approach aimed to evaluate the care pathway’s effectiveness in providing an accurate and thorough assessment of patient conditions from their initial presentation in the ED. Accuracy and completeness were assessed by authors MV and HH, both with appropriate expertise with practical care for older patients. They independently ranked these outcomes scale, and all scores were discussed afterwards until consensus was reached. If consensus could not be reached, MK made the final decision.

We reviewed all items of the STROBE guideline and have included the checklist as supplementary material (see table S4).

### Statistical method

Baseline characteristics of the participants were analysed using descriptive statistics. Continuous data were presented as mean and standard deviation (SD) or median and interquartile range (IQR), and nominal data were presented as total amount and percentage. To compare the baseline characteristics between the control and intervention group, p values were calculated by using Pearson’s Chi-square or Fisher’s exact test for categorical variables, and the independent-samples t test or Mann–Whitney U test for continuous variables. A significance level of 5% was used for all statistical tests. Data of hospitals 1 and 2 were pooled, as the clinical presentation of both groups was similar and reflected daily practice.

Additionally, a post hoc power analysis was conducted to assess the risk of type II errors and evaluate whether non-significant findings could be attributed to limited statistical power. The analysis was performed with a predefined significance level (α = 0.05) and a conventional power threshold of 0.80 to determine the study's capacity to detect meaningful differences.Statistical analyses were performed using the software Statistical Package for the Social Sciences (SPSS), version 27 (IBM Corp, New York, USA). GPower was additionally used for the post hoc power analysis.

## Results

### Hospital and emergency department characteristics

Two hospitals (hospital 1 and 2) successfully implemented the NSC care pathway. Hospital characteristics are presented in Table 1. Hospital 1, a general teaching hospital, provides 24/7 ED service with only occasional presentation stops and includes a co-located GP out-of-hours service. The ED houses 19 operational beds during office hours and 15 operational beds during evenings and nights. Emergency physicians (EP) and internal medicine residents are 24/7 present. Internists are present during office hours and available on call during evenings and nights. Hospital 2, a non-teaching general hospital, experiences a lower annual volume of ED visits and offers 24/7 ED service with infrequent presentation stops. This ED includes four observation beds, supervised by the EP, intended for patient monitoring rather than admission. The EP is present during day and evenings, and during nights on call. Similar to hospital 1, it also features a co-located GP out-of-hours service.

**Table 1:** Hospital and emergency department characteristics of participating hospitals

	Hospital 1	Hospital 2
*Hospital characteristics*		
Hospital type	Teaching general hospital	Non-teaching general hospital
Acute medical care unit, present	Yes	Yes
Hospital admission stop, in a year	22	15
*Emergency department characteristics*		
Number of ED visits per year	20.500	13.500
Number of operational ED beds	8 AM—5PM: 19 ED rooms 4 PM – 8AM; 15 ED-rooms 1 triage room	7 ED rooms 4 observatory beds* 1 triage room
Opening hours	24/7	24/7
Presentation stops ED, in a year	Few	Few
Presence of any internist	Present during office hours; on call during evenings/nights	Present during office hours, on call during evenings/nights
Presence of any geriatrician	Present during office hours. Not on call	Present during office hours, on call during evening/nights
Presence of any emergency physician	24/7	Present during office hours and evenings, on call during nights
Collaboration with the GP out of hours services	Co-located; located in the hospital, but own reception desk and triage procedure	Co-located; located in the hospital, but own reception desk and triage procedure

*Observation beds, located next to the ED, are for monitoring rather than admission, e.g. awaiting diagnostics or observing post-allergic reactions for 4 + hours. Responsibility lies with the ED physician.

### Baseline characteristics

**Fig. 1:**
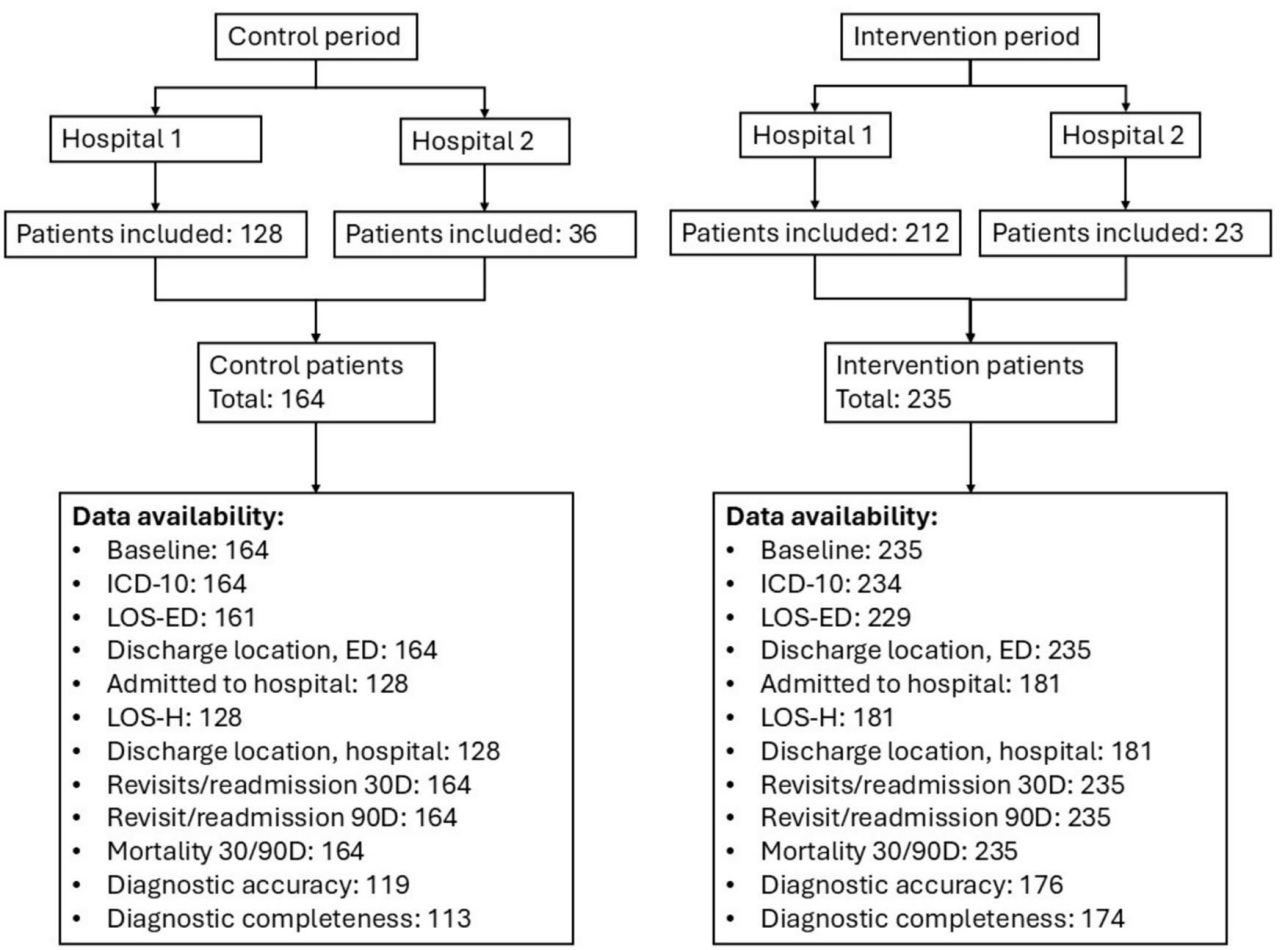
Availability of data. Number of patients are presented in total numbers after ‘:’. LOS-ED: length of stay in the ED. LOS-H: length of stay in hospital. 30/90D: 30- and 90-day follow-up. Accuracy: 100% match between the working diagnosis at ED discharge and hospital discharge. Completeness: 100% match between all diagnoses at ED discharge and hospital discharge

Patients in the control and intervention groups were compared across the two hospitals combined and within each hospital individually. Hospital 1 included 128 control patients between 1–8–2020 and 31–3–2021 and 212 intervention patients between 1–4–2021 and 30–11–2024. Hospital 2 included 36 control patients between 1–12–2020 and 31–8–2021 and 23 intervention patients from 1–9–2021 until 30–11–2024. Figure 1 illustrates data availability for both hospitals.

Baseline characteristics are presented in Table 2 and detailed information on the distribution of all baseline characteristics and outcome measurements can be found in the additional material, Tables S1 and S2. Significant differences were observed between the combined control and intervention groups. Intervention patients used a higher number of medications than control patients (≥ 5 medications 167 (71.1%) vs. 83 (50.6%), *p* < 0.001). Additionally, intervention patients were more likely to live alone at home compared to control patients (56.8% vs 45.5%, *p* = 0.018) and were often assigned lower triage levels (U3–U5 70.4% vs 56.8%, *p* < 0.001). Among those screened with the APOP screener, 69.3% were identified as high risk on cognitive, functional or both domains. Somatic problems were the most frequent reason for referral, affecting 47.7% of intervention patients, followed by cognitive issues at 22.1%.

**Table 2:** Baseline characteristics

	All patients			Hospital 1			Hospital 2		
	Control (*n* = 164)	Intervention (*n* = 235)	*p* value	Control *n* = 128	Intervention *n* = 212	*p* value	Control (*n* = 36)	Intervention (*n* = 23)	*p* value
Age (mean ± SD)	82.9 ± 5.9	83.9 ± 6.3	0.135	82.7 ± 5.9	83.7 ± 6.2	0.134	83.8 ± 6.2	85.2 ± 7.2	0.409
Sex (*n* (%)) Male Female	80 (48.8%) 84 (51.2%)	111 (47.2%) 125 (52.8%)	0.761	62 (48.4%) 66 (51.6%)	97 (45.8%) 115 (54.2%)	0.631	18 (50.0%) 18 (50.0%)	14 (60.9%) 9 (39.1%)	0.414
Number of medication (*n* (%)) 0 1–2 3–4 ≥ 5	29 (17.7%) 26 (15.9%) 26 (15.9%) 83 (50.6%)	7 (3.0%) 20 (8.5%) 41 (17.4%) 167 (71.1%)	< 0.001	29 (22.7%) 23 (18.0%) 20 (15.6%) 56 (43.8%)	6 (2.8%) 19 (9.0%) 34 (16.0%) 153 (72.2%)	< 0.001	0 (0.0%) 3 (8.3%) 6 (16.7%) 27 (75.0%)	1 (4.3%) 1 (4.3%) 7 (30.4%) 14 (60.9%)	0.282
Home situation (*n* (%)) Home, single Home, with partner Home, no further information Care/nursing home Other care institution*	71 (45.5%) 63 (40.4%) 15 (9.6%) 5 (3.2%) 2 (1.3%)	133 (56.8%) 88 (37.6%) 4 (1.7%) 9 (3.8%) 0 (0.0%)	0.001	52 (42.3%) 51 (41.5%) 14 (11.4%) 4 (3.3%) 2 (1.6%)	121 (57.3%) 79 (37.4%) 4 (1.9%) 7 (3.3%) 0 (0.0%)	0.001	19 (57.6%) 12 (36.4%) 1 (3.0%) 1 (3.0%) 0 (0.0%)	12 (52.2%) 9 (39.1%) 0 (0.0%) 2 (8.7%) 0 (0.0%)	0.582
Home care, yes (*n* (%))	50 (38.8%)	113 (48.9%)	0.063	39 (40.6%)	100 (47.8%)	0.240	11 (33.3%)	13 (59.1%)	0.059
Referral (*n* (%)) Self-referred 112 GP/ECP Outpatient department Other care institution	44 (26.8%) 10 (6.1%) 105 (64.0%) 3 (1.8%) 2 (1.2%)	40 (17.0%) 8 (3.4%) 181 (77.0%) 5 (2.1%) 0 (0.0%)	0.025	44 (34.4%) 5 (3.9%) 75 (58.6%) 2 (1.6%) 2 (1.6%)	40 (18.9%) 8 (3.8%) 159 (75.0%) 5 (2.4%) 0 (0.0%)	0.011	0 (0.0%) 5 (13.9%) 30 (83.3%) 1 (2.8%) 0 (0.0%)	0 (0.0%) 0 (0.0%) 22 (95.7%) 1 (4.3%) 0 (0.0%)	0.130
Mode of transportation (*n* (%)) Ambulance Own transport	80 (49.1%) 83 (50.9%)	117 (50.2%) 116 (49.8%)	0.824	70 (54.7%) 58 (45.3%)	106 (50.0%) 106 (50.0%)	0.402	10 (28.6%) 25 (71.4%)	11 (52.4%) 10 (47.6%)	0.075
Specialist in charge (*n* (%)) Emergency physician Internal medicine Geriatrician Other^†^	53 (32.3%) 83 (50.6%) 23 (14.0%) 5 (3.0%)	36 (15.3%) 176 (74.9%) 23 (9.8%) 3 (1.3%)	< 0.001	53 (41.4%) 75 (58.6%) 0 (0.0%) 0 (0.0%)	36 (17.0%) 176 (83.0%) 0 (0.0%) 0 (0.0%)	< 0.001	0 (0.0%) 8 (22.2%) 23 (63.9%) 5 (13.9%)	0 (0.0%) 0 (0.0%) 23 (100.0%) 0 (0.0%)	0.031
Triage code (*n* (%)) U0 U1 U2 U3 U4 U5	1 (0.6%) 11 (6.8%) 58 (35.8%) 82 (50.6%) 4 (2.5%) 6 (3.7%)	0 (0.0%) 6 (2.6%) 63 (27.0%) 134 (57.5%) 14 (6.0%) 16 (6.9%)	0.019	1 (0.8%) 11 (8.7%) 47 (37.3%) 60 (47.6%) 4 (3.2%) 3 (2.4%)	0 (0.0%) 5 (2.4%) 61 (28.8%) 120 (56.6%) 13 (6.1%) 13 (6.1%)	0.008	0 (0.0%) 0 (0.0%) 11 (30.6%) 22 (61.1%) 0 (0.0%) 3 (8.3%)	0 (0.0%) 1 (4.8%) 2 (9.5%) 14 (66.7%) 1 (4.8%) 3 (14.3%)	0.115
APOP screening (*n* (%)) Low risk High risk of functional decline Evidence of cognitive impairment High risk of functional decline and evidence of cognitive impairment	NA	31 (30.7%) 13 (12.9%) 18 (17.8%) 39 (38.6%)	NA	NA	31 (32.3%) 13 (13.5%) 18 (18.8%) 34 (35.4%)	NA	NA	0 (0.0%) 0 (0.0%) 0 (0.0%) 5 (100.0%)	NA
Referral category (*n* (%)) Somatic Higher need for care/social situation Functional status Cognitive problems Inexplicable falling	NA	112 (47.7%) 22 (9.4%) 21 (8.9%) 52 (22.1%) 28 (11.9%)	NA	NA	103 (48.6%) 18 (8.5%) 18 (8.5%) 48 (22.7%) 25 (11.8%)	NA	NA	9 (39.1%) 4 (17.4%) 3 (13.0%) 4 (17.4%) 3 (13.0%)	NA

Data are reported as number of participants (%), mean ± standard deviation or median (Q1-Q3). Percentages were computed relative to the total number of participants in the presented column

*SD* standard deviation. *GP* general practitioner. *ECP* elderly care physician. *APOP* acute presenting older patient. *NA* not applicable

*Hospital 1: other care institution = mental health services

**†**Hospital 2: specialist in charge = 1 neurology, 1 general surgery, 3 pulmonology

When analysed in hospital 1, these trends aligned with the combined results from both hospitals. However, in hospital 2, no differences in the number of medication, home situation or triage levels were found.

### Adherence to the care pathway

Although APOP screening is a prerequisite in the care pathway, only 43.0% of patients underwent screening. As shown in Table 2, there were fewer patients with incomplete information regarding their home situation, which was a part of the structural history taking.

Adherence to diagnostic measurements was high, with laboratory testing exceeding 92.2% and urinanalysis, ECG and chest radiography showing adherence rates above 86.4%. The lowest adherence was observed for bladder ultrasound at 70.4%.

### Outcome measurements

Outcome measurements are presented in Table 3. Among all patients, the median LOS-ED was similar between the control and intervention groups (respectively, 212 (179–252) min vs. 213 (176–261) min, *p* = 0.874). However, among patients discharged directly from the ED, the intervention group had a (non-significant) longer LOS-ED compared to the control group (220 vs. 177 min, *p* = 0.053). Although the majority of the patients were admitted to the hospital in both interventions as the control group, a small percentage of intervention patients could be discharged from the ED immediately to a psychiatric/geriatric crisis ward versus none in the control group (2.1% vs 0.0%, *p* = 0.051). No significant differences were found in LOS-H.

**Table 3:** Outcome measurements

	All patients		*p* value	Hospital 1			Hospital 2		
	Control (*n* = 164)	Intervention (*n* = 235)		Control *n* = 128	Intervention *n* = 212	*p* value	Control (*n* = 36)	Intervention (*n* = 23)	*p* value
Length of stay ED, min (median (Q1–Q3)) All patients Admitted patients Discharged patients	212 (179–252) 213 (187–256) 177 (150–218)	215 (176–261) 212 (176–264) 220 (172–259)	0.874 0.372 0.053	213 (180 – 254) 217 (189–257) 179 (152–218)	213 (177–264) 212 (176–265) 224 (180–261)	0.837 0.219 0.066	197 (176–238) 204 (184–248) 176 (145–210)	222 (168–259) 227 (200–261) 168 (141–235)	0.566 0.523 0.884
Waiting time in ED, min (median (Q1–Q3))	19 (12–34.5)	18 (8–30)	0.058	18.5 (11–31)	18 (8.5–29.5)	0.239	22 (15–41)	18 (5–34)	0.218
Discharge location after ED visit (%)) Own home General ward ICU/MCU Admission to (psychiatric/geriatric) crisis ward	36 (22.0%) 120 (73.2%) 8 (4.9%) 0 (0.0%)	47 (20.0%) 178 (75.7%) 5 (2.1%) 5 (2.1%)	0.051	27 (21.1%) 93% (72.7%) 8 (6.3%) 0 (0.0%)	42 (19.8%) 160 (75.5%) 5 (2.4%) 6 (2.9%)	0.698	9 (25.0%) 27 (75.0%) 0 (0.0%) 0 (0.0%)	5 (21.7%) 18 (78.3%) 0 (0.0%) 0 (0.0%)	0.774
Admitted to hospital (n (%))*	128 (78.0%)	181 (77.0%)	0.809	101 (78.9%)	164 (77.4%)	0.739	27 (75.0%)	17 (73.9%)	0.925
Length of stay in hospital, in days (median (Q1–Q3))* Days until discharge ready Days until actual discharge	4.5 (2–8) 6 (2–10)	5 (2–10) 7 (3–13)	0.308 0.108	4 (2–6.5) 6 (2–9)	5 (2–10) 7 (3–13)	0.043 0.022	7 (4–10.5) 7 (4–13)	5 (2–11) 6 (2–12)	0.316 0.365
Discharge location after admission (*n* (%))* Own home Care/nursing home Rehabilitation Hospice Died in hospital Other^†^	73 (57.0%) 11 (8.6%) 20 (15.6%) 9 (7.0%) 13 (10.2%) 2 (1.6%)	106 (57.3%) 16 (8.6%) 44 (23.8%) 7 (3.8%) 9 (4.9%) 3 (1.6%)	0.219	59 (58.4%) 8 (7.9%) 18 (17.8%) 5 (5.0%) 11 (10.9%) 0 (0.0%)	94 (57.3%) 13 (7.9%) 41 (25.0%) 7 (4.3%) 8 (4.9%) 1 (0.6%)	0.354	14 (51.9%) 3 (11.1%) 2 (7.4%) 4 (14.8%) 2 (7.4%) 2 (7.4%)	11 (55.6%) 2 (11.1%) 3 (16.7%) 0 (0.0%) 1 (5.6%) 2 (11.1%)	0.398
Revisits in 30 days after ED visit (*n* (%))	23 (15.2%)	23 (10.1%)	0.309	16 (13.7%)	20 (9.8%)	0.563	7 (20.6%)	3 (13.6%)	0.724
Readmissions in 30 days after ED visit (*n* (%))	19 (12.7%)	17 (7.5%)	0.265	12 (10.3%)	145 (7.4%)	0.657	7 (20.6%)	2 (9.1%)	0.458
Revisits in 90 days after ED visit (*n* (%))	32 (21.5%)	41 (20.2%)	0.997	22 (19.3%)	34 (18.8%)	0.932	10 (29.4%)	7 (31.8%)	0.469
Readmissions in 90 days after ED visit (*n* (%))	32 (21.5%)	31 (14.5%)	0.172	22 (19.3%)	25 (12.9%)	0.238	10 (29.4%)	6 (27.3%)	0.863
Mortality (*n* (%)) In-hospital mortality 30 day mortality^‡^ 90 day mortality	13 (9.8%) 30 (18.3%) 42 (25.6%)	9 (4.0%) 24 (10.3%) 44 (18.7%)	0.039 0.022 0.149	11 (10.7%) 22 (17.2%) 32 (25.0%)	8 (3.9%) 23 (10.8%) 43 (20.3%)	0.020 0.095 0.345	2 (6.7%) 8 (22.2%) 10 (27.8%)	1 (4.5%) 1 (4.8%)4 (17.3%)	0.743 0.060 0.028

Data are reported as number of participants (%), mean ± standard deviation or median (Q1-Q3). Percentages were computed relative to the total number of participants in the presented column. The 30-day revisits/readmissions/mortality were included within the 90-day revisits/readmissions/mortality

*SD* standard deviation. *NA* not applicable. *ED* emergency department. *ICU* intensive care unit. *MCU* medium care unit

*Percentages were computed relative to the total number of admitted patients

†Hospital 1: intervention = mental health services. Hospital 2: control = shelter and support for the homeless, first-line care stay *n* = 1; intervention = mental health services *n* = 1, first-line care stay *n* = 1

‡Death in hospital was included in the 30 day time frame if it occurred within 30 days of the ED visit

The intervention group showed lower rates of both revisits and readmissions, although neither difference reached statistical significance. The percentage of revisits within 30 days was lower in the intervention group (9.7% vs. 15.2%, *p* = 0.107), and 30 day readmission rate was also lower (7.1% vs. 12.7%, *p* = 0.070). In addition, in-hospital mortality and 30-day mortality were lower in the intervention group compared to control patients (respectively, 4.0% vs 9.8%, *p* = 0.039; 10.3% vs 18.3, *p* = 0.022). These differences appeared to be primarily driven by results from hospital 1, with no significant differences observed in hospital 2. By 90 days, this difference was no longer observed. When comparing outcome measurements within hospital 1 and hospital 2, the results were consistent with the combined data from both hospitals.

Table 4 presents the perceived quality of care, assessed by the PRM-Acute Care, among intervention patients. Out of 235 intervention patients, 116 (49.4%) completed the questionnaire. The primary reason for non-completion was impaired cognitive function during the ED visit. The median symptom relief was rated at 2 out of 10. The median score for understanding the diagnosis was 5 out of 6 (Q1-Q3; 4.0–5.5). Understanding of the treatment plan had a median score of 5 out of 6 (Q1-Q3; 4.0–5.5). Patient experience, reassurance, and perceived quality of care were rated positively, and the average satisfaction rating for the ED visit was 80 out of 10 (8.0–9.0). Results were consistent across both hospitals.

**Table 4:** Perceived quality of care

	Total (*n* = 235)		Hospital 1 (*n* = 212)		Hospital 2 (*n* = 23)	
	*n*	Median (Q1–Q3)	*n*	Median (Q1–Q3)	*n*	Median (Q1–Q3)
Total score domains/perceived quality of care (1–6)	116 (49.4%)	4.3 (3.8–4.9)	102 (48.1%)	4.3 (3.9–4.9)	14 (60.9%)	3.9 (3.4–5.0)
Symptom relief (0–10)	110 (46.8%)	2.0 (2.0–3.0)	97 (45.8%)	2.0 (2.0–3.0)	13 (56.5%)	3.0 (2.0–4.0)
Understanding the diagnosis (0–6)	113 (48.1%)	5.0 (4.0–5.5)	100 (47.2%)	5.0 (4.0–5.8)	13 (56.5%)	4.0 (4.0–5.5)
Understanding the treatment plan (1–6)	114 (48.5%)	5.0 (4.0–5.5)	100 (47.2%)	5.0 (4.5–5.5)	14 (60.9%)	4.5 (3.0–6.0)
Experiences (1–6)	115 (48.9%)	5.3 (5.0–5.8)	101 (47.6%)	5.3 (5.0–5.8)	14 (60.9%)	5.0 (4.5–5.7)
Reassurance (1–6)	111 (47.2%)	5.0 (4.0–6.0)	97 (45.8%)	5.0 (4.0–6.0)	14 (56.5%)	5.0 (4.5–5.7)
Overall satisfaction: What rating would you give the ED on a scale of 0–10? (scale 0–10)	113 (48.1%)	8.0 (8.0–9.0)	100 (47.6%)	8.0 (8.0–9.0)	13 (56.5%)	8.0 (7.5–9.0)

Data are reported as the number of participants (%) and median (Q1–Q3). Percentages were computed relative to the total number of participants in the presented column

Table 5 presents the accuracy and completeness of diagnoses for admitted patients. A total of 128 patients in the control group and 181 patients in the intervention group were admitted. Due to missing conclusions in letters from the ED or hospital admission, the analysis included 119 patients in the control group (93.0%) and 176 patients in the intervention group (96.7%). Consensus between MV and HH was reached in nearly all cases, with MK required to make the final decision in only two instances. This rare occurrence highlights the high level of agreement between the two assessors.

**Table 5:** Accuracy and completeness of diagnoses

	Control (*n* = 119)	Intervention (*n* = 176)	*p* value
Accuracy of working diagnosis	82/119 (68.9%)	136/176 (76.8%)	0.129
Completeness of diagnoses	42/113 (37.2%)	82/174 (47.4%)	0.096

Data are reported as the number of participants (%)

Both accuracy and completeness were measured as either complete or not complete. The intervention group showed a higher percentage in both metrics: 76.8% for accuracy compared to 68.9% in the control group (p = 0.129), and 47.4% for completeness compared to 37.2% in the control group (p = 0.096). However, neither of these differences reached statistical significance. An overview of the diagnoses registered following the ICD-10 classification is provided in the additional materials, Table S3. No significant differences were observed in the types of diagnosis according to the ICD-10 classification between the intervention and control groups.

### Post hoc power analysis

To assess the risk of a type II error due to the limited sample size, we conducted a post hoc power analysis. The effect size (Cohen’s d) was -0.03, indicating a minimal difference between groups. With a pooled standard deviation of 67.05, the analysis yielded a power of 0.08, which is well below the conventional threshold of 0.80. This suggests a high probability of failing to detect a true effect, even if one exists.

## Discussion

In this study, implementation of the NSC care pathway in two hospitals provided valuable insights into the outcomes for patients included in the NSC care pathway. We showed that patients with NSC are often frail, as identified by the APOP screener, with high rates of home care arrangements and polypharmacy. These characteristics are associated with adverse outcomes, emphasising the need for a comprehensive assessment at the ED [21, 22]. The NSC care pathway was designed to address this need.

Although the NSC care pathway showed promising results, it did not lead to a reduction in the primary outcome of LOS-ED. Specifically, the LOS-ED for admitted patients in the intervention group was similar to that of the control group, but longer for patients who were discharged. A small number of patients were admitted to psychiatric or geriatric units outside the hospital, and due to logistical challenges, this took several hours. However, even when these cases are considered outliers, the overall result remains unchanged.

Several factors may explain the lack of improvement of LOS-ED. While the pathway facilitated comprehensive history taking and standardised diagnostic testing—which in itself could take more time—it did not address other critical factors impacting ED throughput. These factors include overcrowding, the prioritisation of more urgent cases, delays in laboratory results and imaging bottlenecks caused by competing requests. Moreover, the care pathway does not address ongoing logistical challenges, including shortages of hospital beds and staff, yet those significantly impact measurable outcomes like LOS-ED [23–26]. The participation of only two hospitals, instead of the expected six, along with the mid-study withdrawal of a third hospital and the impossibility to appoint an ED coach, highlights the challenges faced by acute care settings, which are often burdened by time constraints, high patient volumes and staffing pressures [23, 24].

While LOS-ED was chosen as the primary outcome, its relevance can be debated. From a patient-centred perspective, length of stay in the ED may not be the most meaningful outcome, as comfort, perceived quality of care and diagnostic accuracy may be more important. Additionally, the focus on LOS-ED is primarily driven by the need to optimise efficiency and patient flow in the ED. However, in some cases, a longer ED stay may be necessary to ensure a thorough diagnostic process and appropriate clinical decision-making. Given these considerations, future evaluations of the care pathway should incorporate patient-reported outcomes and measures of clinical effectiveness, such as preservation of functional status, quality of life and mortality, as primary outcomes alongside LOS-ED to provide a more comprehensive assessment of its impact.

In contrast to the limited impact on LOS-ED and LOS-H, intervention patients showed lower rates of 30-day readmissions and a significant reduction in both in-hospital and 30-day mortality. Furthermore, the intervention demonstrated non-significant trends towards improved diagnostic accuracy and completeness. This may suggest that the pathway led to a better understanding of patients' conditions, potentially explaining the lower rates of 30-day revisits and readmissions. However, the limited effect on diagnostic completeness could be attributed to the way it was measured. The reliance on discharge letters as a source for completeness could have led to incomplete or inconsistent documentation of all relevant diagnostic information.

Additionally, the perceived quality of care was rated positively in areas such as symptom relief, understanding of diagnosis and treatment plans, and overall care experience. However, it is important that symptom relief was rated lower in our study compared to the validation study of the PRM acute care [18]. This may be due to the nature of NSC, which commonly cannot be fully addressed in the limited time available in the ED. Among the 235 intervention patients, 116 (49.4%) completed the questionnaire. A significant barrier to completion was impaired cognitive function, as 22.1% of patients exhibited cognitive problems during their ED visit. Notably, the PRM acute care was validated for patient self-reporting only, not for proxy respondents such as informal caregivers. This limitation underscores the need for future studies to consider the use of proxy reports when cognitive impairment may hinder self-reporting, ensuring that patients with cognitive challenges are not excluded from these evaluations. Improving both diagnostic accuracy and completeness, while maintaining high quality of care, provides a strong foundation for patient-centred care. By promoting a clear understanding of patients' needs and preferences, the pathway encourages shared decision-making and may lead to more personalised care.

Another key observation in this study is the assignment of lower urgency categories to intervention patients, which may explain their longer LOS-ED and could suggest potential under-triage. Despite the fact that NSC can mask serious underlying conditions, many intervention patients were categorised as lower urgency [3, 8, 27]. Under-triage is a known issue for older adults in urgent care settings, with reported rates ranging from 25.3% to 61.3% [6, 28, 29]. It remains uncertain whether the intervention group was potentially different than the retrospectively composed control group and if their NSC were potentially ‘less non-specific’ leading to higher urgency scores or if they experienced less under-triage. It is known that patients with NSC show higher than expected mortality rates based on triage systems [30]. However, it is unclear if faster treatment in the ED would directly influence these outcomes, as the underlying frailty may be a more significant factor than time-critical conditions. This raises the question of whether reducing LOS-ED would result in improved outcomes for this patient population.

Additionally, the higher rate of polypharmacy in the intervention group, a common characteristic among older adults with NSC, could complicate clinical assessments and decision-making, potentially influencing triage decisions and subsequent outcomes. Polypharmacy increases the risk of adverse drug reactions, complications and misdiagnosis, which may contribute to longer hospital stays and higher mortality rates [31]. These baseline differences may have confounded the relationship between the care pathway and clinical outcomes.

Protocol adherence was not specifically monitored, which may have led to variations in implementation and contributed to inconsistencies. While adherence to diagnostic tests was generally high, frailty screening rates were lower than expected. This could be due to the lack of a dedicated ED nurse, potentially resulting in missed or delayed assessments, which may have reduced the pathway's effectiveness and limited its impact on outcomes such as LOS-ED and patient satisfaction.

### Limitations

The study faced limitations due to its design, particularly in control group selection. The variation in selection method may have caused baseline differences between the intervention and control group. The control group had fewer cases of polypharmacy, higher urgency codes and more intensive and medium care unit (ICU and MCU) admissions, suggesting they were less frail but more acutely ill [32–34]. The control’s shorter LOS-ED may reflect the need for rapid ICU/MCU transfer, potentially biasing LOS-ED results. Furthermore, the prospective inclusion of control patients in hospital 2 may have introduced selection bias, as prior knowledge of their inclusion could have influenced the initial care provided to these patients. Additionally, due to the study design, no pre–post comparisons of patient-reported outcomes were available, and intervention adherence was limited, particularly among cognitively impaired patients. As a result, differences in the effect of the care pathway could not be demonstrated. Last, there was a risk for selection bias, as inclusion relied on specialist judgement, leading to inconsistencies across hospitals. While these limitations introduce potential confounders, they highlight the realities of clinical practice, where variations in physician experience, patient selection and evolving protocols are inevitable.

Another important limitation is the lower than expected sample size. The pandemic likely influenced hospital willingness to participate, as only two hospitals continued the study instead of the anticipated six. Additionally, hospital 2 experienced a low inclusion rate due to logistical challenges, which affected systemic identification and enrolment of eligible patients, further limiting the sample size. The post hoc power analysis indicates a high probability of failing to detect a true effect, even if one existed. As a result, the reduced sample size may have limited the study’s ability to identify statistically significant differences and could affect the generalisability of the findings. The low study power also suggests that the study may not have been adequately equipped to detect meaningful effects, reinforcing the need for future studies with larger, more balanced cohorts.

### Future research

Future research should refine the NSC care pathway by improving protocol adherence and optimising study design, aiming for well-balanced patient inclusion. Alternative methods like propensity score matching or well-designed cohort studies could better correct for confounding variables. Monitoring adherence through audits and dedicated ED coordinators may enhance protocol fidelity and result reliability.

Collecting data on contextual factors, such as overcrowding and staffing shortages potentially affecting the effects of the NSC care pathway, could help interpret LOS-ED, LOS-H and patient outcomes. Incorporating patient-reported outcomes, including proxy reports for cognitively impaired patients, would provide valuable insights into care quality. Evaluating included diagnostic tests could improve the cost-effectiveness and accuracy.

Larger sample sizes are needed to detect differences in mortality, readmissions and length of stay. Future studies should prioritise patient-centred outcome measures, such as functionality and quality of life, while addressing logistical barriers to improve cohort balance and generalisability. A focus on patient-centred care, real-world feasibility and continuous process improvement will help optimise the NSC care pathway for older adults.

## Conclusions

This study is the first to implement an NSC care pathway in the ED, showing trends towards improved diagnostic accuracy and completeness. The frailty and complex needs of NSC patients highlight the need for effective management, but mixed results reveal implementation challenges.

## Supplementary Information

Below is the link to the electronic supplementary material.

Supplementary file1 (DOCX 37 KB)

## Data Availability

Data are available upon reasonable request by contacting the corresponding author.
