# Supplementary material for: Evaluation of a care pathway for older adults presenting with nonspecific complaints at the emergency department: a before-and-after study
Source: Eur Geriatr Med. 2025 May 7;16(4):1537–49. doi: 10.1007/s41999-025-01226-8 (PMC12378488; doi:10.1007/s41999-025-01226-8)
Supplement: Supplementary file 1 — Supplementary file1 (DOCX 37 KB) [file 41999_2025_1226_MOESM1_ESM.docx]

**Table S1. Distribution of baseline characteristics and statistical tests used**

|  | All patients | | Hospital 1 |  | Hospital 2 |  |
| --- | --- | --- | --- | --- | --- | --- |
|  | **Distribution** | **Statistical test used** | **Distribution** | **Statistical test used** | **Distribution** | **Statistical test used** |
| Age | Normal | Independent t-test | Normal | Independent t-test | Normal | Independent t-test |
| Sex | Categorical | Pearson chi-square | Categorical | Pearson chi-square | Categorical | Pearson chi-square |
| Number of medicines | Categorical | Pearson chi-square | Categorical | Pearson chi-square | Categorical | Fisher’s exact |
| Home situation | Categorical | Pearson chi-square | Categorical | Fisher’s exact | Categorical | Fisher’s exact |
| Home care, yes | Categorical | Pearson chi-square | Categorical | Pearson chi-square | Categorical | Pearson chi-square |
| Referrer | Categorical | Fisher’s exact | Categorical | Fisher’s exact | Categorical | Fisher’s exact |
| Mode of transportation | Categorical | Pearson chi-square | Categorical | Pearson chi-square | Categorical | Pearson chi-square |
| Specialist in charge | Categorical | Pearson chi-square | Categorical | Pearson chi-square | Categorical | Fisher’s exact |
| Triage code | Categorical | Fisher’s exact | Categorical | Fisher’s exact | Categorical | Fisher’s exact |
| APOP screening | Categorical | NA | Categorical | NA | Categorical | NA |
| Referral category | Categorical | NA | Categorical | NA | Categorical | NA |

*APOP: Acute Presenting Older Patient. NA: not applicable.*

**Table S2. Distribution of outcome measurements and statistical tests used**

|  | All patients | |  | Hospital 1 |  | Hospital 2 |  |
| --- | --- | --- | --- | --- | --- | --- | --- |
|  | **Distribution** | | **Statistical test used** | **Distribution** | **Statistical test used** | **Distribution** | **Statistical test used** |
| Length of stay ED  All patients  Admitted patients  Discharged patient | Non-normal  Non-normal  Normal | | Mann-Whitney U Mann-Whitney U  Mann-Whitney U for consistency | Non-normal  Non-normal  Normal | Mann-Whitney U Mann-Whitney U  Mann-Whitney U for consistency | Non-normal  Non-normal  Non-normal | Mann-Whitney U Mann-Whitney U  Mann-Whitney U |
| Waiting time in ED | Non-normal | | Mann-Whitney U | Non-normal | Mann-Whitney U | Non-normal | Mann-Whitney U |
| Discharge location after ED-visit | Categorical | | Fisher’s exact | Categorical | Fisher’s exact | Categorical | Pearson chi-square |
| Admitted to hospital | Categorical | | Pearson chi-square | Categorical | Pearson chi-square | Categorical | Pearson chi-square |
| Length of stay hospital  Discharge ready  Actual discharge | | Non-normal  Non-normal | Mann-Whitney U Mann-Whitney U | Non-normal  Non-normal | Mann-Whitney U Mann-Whitney U | Non-normal  Non-normal | Mann-Whitney U Mann-Whitney U |
| Discharge location after admission | Categorical | | Fisher’s exact | Categorical | Fisher’s exact | Categorical | Fisher’s exact |
| Revisits in 30 days after ED-visit | Categorical | | Fisher’s exact | Categorical | Fisher’s exact | Categorical | Fisher’s exact |
| Readmissions in 30 days after ED-visit | Categorical | | Fisher’s exact | Categorical | Fisher’s exact | Categorical | Fisher’s exact |
| Revisits in 90 days after ED-visit | Categorical | | Fisher’s exact | Categorical | Fisher’s exact | Categorical | Fisher’s exact |
| Readmissions in 90 days after ED-visit | Categorical | | Fisher’s exact | Categorical | Fisher’s exact | Categorical | Pearson chi-square |
| Mortality  In-hospital mortality  30-day mortality  90-day mortality | Categorical  Categorical  Categorical | | Pearson chi-square Pearson chi-square  Pearson chi-square | Categorical  Categorical  Categorical | Pearson chi-square Pearson chi-square  Pearson chi-square | Categorical  Categorical  Categorical | Fisher’s exact Fisher’s exact Fisher’s exact |
| Accuracy of working diagnosis | Categorical | | Pearson chi-square | NA | NA | NA | NA |
| Comprehensiveness of diagnosis | Categorical | | Pearson chi-square | NA | NA | NA | NA |
| Perceived quality of care | Non-normal | | NA | Non-normal | NA | Normal; median (IQR) presented for consistency | Normal; median (IQR) presented for consistency |
| Symptom relief | Non-normal | | NA | Non-normal | NA | Normal; median (IQR) presented for consistency | Normal; median (IQR) presented for consistency |
| Understanding the diagnosis | Non-normal | | NA | Non-normal | NA | Normal; median (IQR) presented for consistency | Normal; median (IQR) presented for consistency |
| Understanding treatment plan | Non-normal | | NA | Non-normal | NA | Normal; median (IQR) presented for consistency | Normal; median (IQR) presented for consistency |
| Experiences | Non-normal | | NA | Normal; median (IQR) presented for consistency | NA | Non-normal | NA |
| Reassurance | Non-normal | | NA | Normal; median (IQR) presented for consistency | NA | Non-normal | NA |
| Overall experience | Normal; median (IQR) presented for consistency | | NA | Normal; median (IQR) presented for consistency | NA | Normal; median (IQR) presented for consistency | Normal; median (IQR) presented for consistency |

ED: Emergency Deparmtent. NA: not applicable.

**Table S3. ICD-10 codes**

|  | All patients  (n=399) | Control  (n=164) | Intervention  (n=235) | p-  value | Statistical test used |
| --- | --- | --- | --- | --- | --- |
| Certain infectious and parasitic diseases | 28 (7.0%) | 13 (8.2%) | 15 (6.5%) | 0.519 | Pearson chi-square |
| Neoplasms | 21 (5.3%) | 8 (5.0%) | 13 (5.6%) | 0.805 | Pearson chi-square |
| Diseases of the blood and blood-forming organs and certain disorders involving the immune mechanism | 11 (2.8%) | 5 (3.1%) | 6 (2.6%) | 0.763 | Fisher’s exact |
| Endocrine, nutritional and metabolic diseases | 17 (4.3%) | 8 (5.0%) | 9 (3.9%) | 0.583 | Pearson chi-square |
| Mental and behavioral disorders | 40 (10.1%) | 15 (9.4%) | 25 (10.8%) | 0.667 | Pearson chi-square |
| Diseases of nervous system | 7 (1.8%) | 2 (1.3%) | 5 (2.2%) | 0.706 | Fisher’s exact |
| Diseases of circulatory system (incl. cerebrovascular accident) | 33 (8.3%) | 12 (7.5%) | 21 (9.1%) | 0.599 | Pearson chi-square |
| Diseases of respiratory system | 30 (7.5%) | 10 (6.3%) | 20 (8.6%) | 0.395 | Pearson chi-square |
| Diseases of the digestive system | 11 (2.8%) | 3 (1.9%) | 8 (3.4%) | 0.536 | Fisher’s exact |
| Diseases of skin and subcutaneous tissue | 4 (1.0%) | 0 (0.0%) | 4 (1.7%) | 0.149 | Fisher’s exact |
| Diseases of the musculoskeletal system and connective tissue | 15 (3.8%) | 3 (1.9%) | 12 (5.2%) | 0.097 | Pearson chi-square |
| Diseases of the genitourinary system | 54 (13.6%) | 27 (17.0%) | 27 (11.6%) | 0.133 | Pearson chi-square |
| Symptoms, signs and abnormal clinical and laboratory findings, not elsewhere classified | 78 (19.6%) | 31 (19.5%) | 47 (20.3%) | 0.853 | Pearson chi-square |
| Injury, poisoning and certain other consequences of external causes | 15 (3.8%) | 7 (4.4%) | 8 (3.4%) | 0.629 | Pearson chi-square |
| Codes for special purposes (incl. COVID) | 15 (3.8%) | 8 (5.0%) | 7 (3.0%) | 0.308 | Pearson chi-square |
| Factors influencing health status and contact with health services. | 12 (3.1%) | 7 (4.4%) | 5 (2.2%) | 0.240 | Fisher’s exact |

**Table S4. STROBE Statement—checklist of items that should be included in reports of observational studies**

|  | Item No. | Recommendation | Page  No. |
| --- | --- | --- | --- |
| **Title and abstract** | 1 | (*a*) Indicate the study’s design with a commonly used term in the title or the abstract | Titlepage + 1 |
|  |  | (*b*) Provide in the abstract an informative and balanced summary of what was done and what was found | 1-2 |
| Introduction | | | |
| Background/rationale | 2 | Explain the scientific background and rationale for the investigation being reported | 3-4 |
| Objectives | 3 | State specific objectives, including any prespecified hypotheses | 4 |
| Methods | | | |
| Study design | 4 | Present key elements of study design early in the paper | 4-5 |
| Setting | 5 | Describe the setting, locations, and relevant dates, including periods of recruitment, exposure, follow-up, and data collection | 4-5 |
| Participants | 6 | (*a*) *Cohort study*—Give the eligibility criteria, and the sources and methods of selection of participants. Describe methods of follow-up | 4-5 |
|  |  | (*b*) *Cohort study*—For matched studies, give matching criteria and number of exposed and unexposed | 4-5 |
| Variables | 7 | Clearly define all outcomes, exposures, predictors, potential confounders, and effect modifiers. Give diagnostic criteria, if applicable | 5-6 |
| Data sources/ measurement | 8 | For each variable of interest, give sources of data and details of methods of assessment (measurement). Describe comparability of assessment methods if there is more than one group | 6+additional material |
| Bias | 9 | Describe any efforts to address potential sources of bias | 4-5 |
| Study size | 10 | Explain how the study size was arrived at | Reference to protocol study, 4 |

Continued on next page

| Quantitative variables | 11 | Explain how quantitative variables were handled in the analyses. If applicable, describe which groupings were chosen and why | 6-7 |
| --- | --- | --- | --- |
| Statistical methods | 12 | (*a*) Describe all statistical methods, including those used to control for confounding | 6-7 |
|  |  | (*b*) Describe any methods used to examine subgroups and interactions | 6-7 |
|  |  | (*c*) Explain how missing data were addressed | NA |
|  |  | (*d*) *Cohort study*—If applicable, explain how loss to follow-up was addressed | NA |
|  |  | (*e*) Describe any sensitivity analyses | NA |
| Participants | 13 | (a) Report numbers of individuals at each stage of study—eg numbers potentially eligible, examined for eligibility, confirmed eligible, included in the study, completing follow-up, and analysed | 9 |
|  |  | (b) Give reasons for non-participation at each stage | NA |
|  |  | (c) Consider use of a flow diagram | 9 |
| Descriptive data | 14 | (a) Give characteristics of study participants (eg demographic, clinical, social) and information on exposures and potential confounders | 9-10 |
|  |  | (b) Indicate number of participants with missing data for each variable of interest | 9 |
|  |  | (c) *Cohort study*—Summarise follow-up time (eg, average and total amount) | 9 |
| Outcome data | 15 | *Cohort study*—Report numbers of outcome events or summary measures over time | *9* |
| Main results | 16 | (*a*) Give unadjusted estimates and, if applicable, confounder-adjusted estimates and their precision (eg, 95% confidence interval). Make clear which confounders were adjusted for and why they were included | NA |
|  |  | (*b*) Report category boundaries when continuous variables were categorized | NA |
|  |  | (*c*) If relevant, consider translating estimates of relative risk into absolute risk for a meaningful time period | NA |

Continued on next page

| Other analyses | 17 | Report other analyses done—eg analyses of subgroups and interactions, and sensitivity analyses | 7, 12 |
| --- | --- | --- | --- |
| Key results | 18 | Summarise key results with reference to study objectives | 12-14 |
| Limitations | 19 | Discuss limitations of the study, taking into account sources of potential bias or imprecision. Discuss both direction and magnitude of any potential bias | 14-16 |
| Interpretation | 20 | Give a cautious overall interpretation of results considering objectives, limitations, multiplicity of analyses, results from similar studies, and other relevant evidence | 12-17 |
| Generalisability | 21 | Discuss the generalisability (external validity) of the study results | 12-17 |
| **Other information** | |  |  |
| Funding | 22 | Give the source of funding and the role of the funders for the present study and, if applicable, for the original study on which the present article is based | 18 |

Note: An Explanation and Elaboration article discusses each checklist item and gives methodological background and published examples of transparent reporting. The STROBE checklist is best used in conjunction with this article (freely available on the Web sites of PLoS Medicine at http://www.plosmedicine.org/, Annals of Internal Medicine at http://www.annals.org/, and Epidemiology at http://www.epidem.com/). Information on the STROBE Initiative is available at www.strobe-statement.org.
